# Development of a Lentiviral Vector for High-Yield Production of Synthetic and Recombinant GCase for Gaucher Disease Therapy

**DOI:** 10.3390/ijms26157089

**Published:** 2025-07-23

**Authors:** Ana Carolina Coelho, Claudia Emília Vieira Wiezel, Alline Cristina de Campos, Lílian Louise Souza Figueiredo, Gabriela Aparecida Marcondes Suardi, Juliana de Paula Bernardes, Daniela Pretti da Cunha Tirapelli, Vitor Marcel Faça, Kuruvilla Joseph Abraham, Carlos Gilberto Carlotti-Júnior, Velia Siciliano, Ron Weiss, Stanton Gerson, Aparecida Maria Fontes

**Affiliations:** 1 Department of Genetics, Ribeirão Preto Medical School, University of São Paulo, Ribeirão Preto 14049-900, SP, Brazil; anacoelhobio@gmail.com (A.C.C.); cwiezel@fmrp.usp.br (C.E.V.W.); lilian.figueiredo@usp.br (L.L.S.F.); gabisuardi@usp.br (G.A.M.S.); julianabernardes@usp.br (J.d.P.B.); 2 Department of Pharmacology, Ribeirão Preto Medical School, University of São Paulo, Ribeirão Preto 14049-900, SP, Brazil; allinecampos@usp.br; 3 Department of Surgery and Anatomy, Ribeirão Preto Medical School, University of São Paulo, Ribeirão Preto 14049-900, SP, Brazil; daniela@fmrp.usp.br; 4 Department of Biochemistry and Immunology, Ribeirão Preto Medical School, University of São Paulo, Ribeirão Preto 14049-900, SP, Brazil; vitor.faca@fmrp.usp.br; 5 Department of Computer Science, Institute of Mathematics and Computer Science, University of São Paulo, São Carlos 13566-590, SP, Brazil; abraham@fmrp.usp.br; 6 Division of Neurology, São Paulo Medical School, University of São Paulo, São Paulo 05403-000, SP, Brazil; carlotti@fmrp.usp.br; 7 Synthetic and Systems Biology Laboratory for Biomedicine, Istituto Italiano di Tecnologia-IIT, Largo Barsanti e Matteucci, 80125 Naples, Italy; velia.siciliano@iit.it; 8 Synthetic Biology Center, Department of Biological Engineering, Massachusetts Institute of Technology, Cambridge, MA 02129-4307, USA; rweiss@mit.edu; 9 School of Medicine, University Hospital Medical Center, Case Western Reserve University, Cleveland, OH 44106, USA; slg5@case.edu

**Keywords:** Gaucher disease, glucocerebrosidase, 293FT cells, codon optimization, hEF1α promoter, lentiviral vector

## Abstract

Gaucher disease (GD) is an autosomal recessive disorder caused by the deficient activity of the lysosomal enzyme glucocerebrosidase (GCase). Although enzyme replacement therapy (ERT) remains the standard of care for non-neuropathic GD patients, its high cost significantly limits accessibility. To enhance production efficiency, we developed a lentiviral system encoding a codon-optimized GCase gene driven by the human elongation factor 1a (hEF1α) promoter for stable production in human cell lines. A functional lentiviral vector, LV_EF1α_GBA_Opt, was generated at a titer of 7.88 × 10^8^ LV particles/mL as determined by qPCR. Six transduction cycles were performed at a multiplicity of infection of 30–50. The transduced heterogeneous human cell population showed GCase-specific activity of 307.5 ± 53.49 nmol/mg protein/h, which represents a 3.21-fold increase compared to wild-type 293FT cells (95.58 ± 16.5 nmol/mg protein/h). Following single-cell cloning, two clones showed specific activity of 763.8 ± 135.1 and 752.0 ± 152.1 nmol/mg/h (clones 15 and 16, respectively). These results show that codon optimization, a lentiviral delivery system, and clonal selection together enable the establishment of stable human cell lines capable of producing high levels of biologically active, synthetic recombinant GCase in vitro. Further studies are warranted for the functional validation in GD patient-derived fibroblasts and animal models.

## 1. Introduction

Gaucher disease (GD) is an autosomal recessive genetic disorder caused by the deficient activity of the lysosomal enzyme glucosylceramidase (GCase), which catalyzes the hydrolysis of glucosylceramide (GlcCer) into ceramide and glucose [1,2,3]. This enzymatic deficiency leads to the pathological accumulation of GlcCer in cells, primarily in macrophages, which induces progressive damage to multiple organs [4,5,6,7]. GD is the most common lysosomal storage disease [8] and presents heterogeneous clinical symptomatology ranging from non-neuropathic (Type 1) to present and severe neurological pathology (GD Type 2 and 3) [9,10,11,12,13].

Currently, enzyme replacement therapy (ERT) remains the standard of care for non-neuropathic forms of GD patients, with well-established efficacy in reversing visceral and hematological manifestations such as hepatosplenomegaly and cytopenia [3,14,15,16]. However, its high cost and limited efficacy in neuronopathic forms restrict its broader applicability. In this context, substrate reduction therapy (SRT), particularly with the orally administered glucosylceramide synthase inhibitor eliglustat, has emerged as an alternative option for selected patients with type 1 GD, offering favorable clinical outcomes and greater convenience [17,18].

As a pioneer in lysosomal storage disease treatment, GD was the first to receive FDA-approved ERT, initially using alglucerase, a placental tissue-derived glucocerebrosidase [19]. To monitor therapeutic outcomes and disease progression, the International Collaborative Gaucher Group (ICGG) established the Gaucher Registry (ClinicalTrials.gov ID NCT00358943, 1991), which collects real-world data on ERT efficacy, safety, and the natural history of GD [3,20,21,22,23].

In 1995, imiglucerase, produced in Chinese hamster ovary (CHO) cells, was the first recombinant human glucocerebrosidase developed [24,25]. Subsequently, velaglucerase alfa, an enzyme expressed in human cell culture systems received approval [26,27]. Later, taliglucerase alfa, a plant-derived recombinant enzyme, was introduced as an emergency treatment to ensure therapy continuity for patients, following viral contamination issues in imiglucerase production [28,29].

In Brazil, these therapies are accessible through the Unified Health System (SUS) under Ordinance No. 1.266 (14 November 2014) [23,30].

The high production cost of recombinant GCase continues to limit the accessibility of enzyme replacement therapy (ERT), driving the need for optimized expression platforms. To address this issue, alternative production strategies have been developed to enhance yield, stability, and tissue-specific delivery. These include viral vector-based approaches using retroviral [31], lentiviral [32], and AAV-mediated targeting of the CCR5 locus [33]. Also, mammalian cell systems make use of methotrexate (MTX) for clone selection and amplification [34] and plant-based platforms [35,36], among other innovative approaches.

Using synonymous codons is a promising strategy to improve human mRNA stability [37] and enhance recombinant protein production [38,39]. This approach has been successfully implemented to increase expression in various genetic diseases, including hemophilia A and B [40,41] and X-linked severe combined immunodeficiency [42], as well as in lysosomal storage disease such as aspartylglucosaminidase [43], Fabry disease [44], Gaucher disease [45,46], mucopolysaccharidosis II [47,48], Krabbe disease [49,50], Pompe disease [51,52,53], Tay–Sachs disease, and Sandhoff disease [54], among others.

Lentiviral vectors (LVs) have been successfully investigated in ex vivo gene therapy for multiple genetic diseases, including applications using patient-derived induced pluripotent stem cells (iPSCs). Clinical and preclinical studies demonstrate their efficacy in hemophilia A [41], X-linked severe combined immunodeficiency [55], Fabry disease [56], Krabbe disease [50,57], mucopolysaccharidosis II [58], and Pompe disease [59], among others. For GCase production, LV systems offer distinct advantages such as efficient transduction of both dividing and non-dividing cells [60,61,62,63], unlike oncoretroviral vectors. Also, unlike oncoretroviral vectors, LV integration sites are preferably in euchromatin regions, which increases the chances to obtain active transgene expression [64,65,66].

In our previous study [46], we demonstrated the feasibility of transient GCase production using codon-optimized GBA-1 cDNA under the hEF1α promoter in 293FT cells. The engineered variant (GBA-Opt) exhibited a 5.2-fold increase in mRNA expression and a 6.1-fold increase in enzymatic activity compared to controls, validating the potential of synthetic biology approaches for ERT [46]. Here, we extend this work by developing a scalable producer cell line through lentiviral genomic integration and clonal selection. Compared to wild-type 293FT controls (7.037 ± 0.4 nmol hydrolyzed substrate/mL/h), our optimized system achieved a 97-fold increase in secreted activity (683.9 nmol/mL/h) in clone 16, along with high specific activity (752.0 ± 152.1 nmol hydrolyzed substrate/mg/h).

## 2. Results

### 2.1. Production of Stable Lentiviral Transduced Human Cell Lines

Lentiviral particles encoding the codon-optimized human *GBA1* gene (GBA-Opt), under the control of the hEF1α promoter (LV-EF1α_GBA-Opt), were produced and concentrated. Particle formation was confirmed by ELISA for the p24 capsid antigen, and the viral titer was determined by quantitative PCR (qPCR), yielding 7.88 × 10^8^ viral particles per mL.

293FT cells were transduced in six successive cycles using different volumes (2, 5, and 10 μL) of the viral preparation, corresponding to multiplicity of infection (MOI) of 30–50. Following transduction, cells were cultured in 2 μg/mL puromycin for 10 days to select a stable heterogeneous puromycin-resistant population (L17_293FT_GBA_OPT_HP). This population was subsequently expanded under low (1 μg/mL) and high (5 μg/mL) puromycin concentrations to enrich for subpopulations with increased expression, prior to GCase activity analysis.

### 2.2. GCase Activity in Lentiviral Transduced and Puromycin-Selected L17_293FT_GBA_OPT_HP Cells

**Figure 1:**
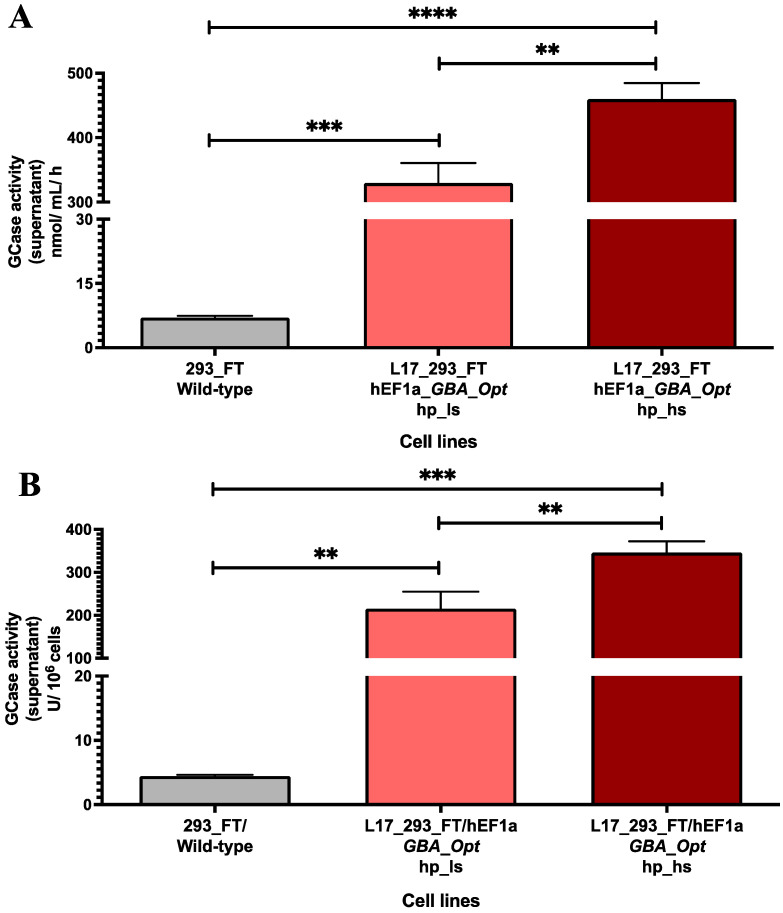
GCase activity in L17_293FT_GBA_OPT_HP cell supernatants following puromycin selection. (**A**) Enzymatic activity (nmol hydrolyzed substrate per mL per h) significantly increased upon puromycin treatment. Treatment with 1 μg/mL puromycin (low stringency, ls) 329.7 ± 31.02 nmol/mL/h of GCase activity, while 5 μg/mL (high stringency, hs) increased activity to 459.7 ± 24.82 nmol/mL/h. Compared to the untreated 293FT/wild-type control, GCase activity increased 47-fold in ls (*p* = 0.0002; t = 20.81, df = 3.001; *n* = 4) and 62.4-fold in hs (*p* < 0.0001; t = 36.48, df = 3.002; *n* = 4). The comparison between hs and ls showed a 1.4-fold increase in activity (*p* = 0.0007; t = 6.547, df = 5.725; *n* = 4). (**B**) GCase activity normalized per million cells (U GCase/10^6^ cells) was also enhanced by puromycin selection. The hp_ls population exhibited 215.4 ± 39.46 U/10^6^ cells, while hp_hs reached 346.1 ± 26.27 U/10^6^ cells. Compared to 293FT/wild-type cells (4.44 ± 0.21 U/10^6^ cells), this corresponds to 48.5-fold (*p* = 0.0017; t = 10.69, df = 3.000; *n* = 4) and 78-fold (*p* = 0.0001; t = 26.01, df = 3.000; *n* = 4) increases, respectively. The comparison between hp_hs and hp_ls showed a 1.6-fold increase (*p* = 0.0023; t = 5.515, df = 5.223; *n* = 4). Statistical analysis was performed using a two-tailed unpaired *t*-test with Welch’s correction. ** indicates *p* ≤ 0.01, *** indicates *p* ≤ 0.001, **** indicates *p* ≤ 0.0001. [293FT: human embryonic kidney cells expressing SV40 large T antigen]; [hEF1α: human elongation factor 1-alpha promoter]; [GBA-Opt: codon-optimized human GBA (glucocerebrosidase) cDNA sequence]; [hp_ls: heterogeneous population under low-stringency puromycin selection (1 µg/mL)]; [hp_hs: heterogeneous population under high-stringency puromycin selection (5 µg/mL)]; [GCase: β-glucocerebrosidase enzyme].

The GCase activity was quantified in the supernatants of L17_293FT_GBA_OPT_HP cells following puromycin selection at low (1 μg/mL) and high (5 μg/mL) stringency concentrations. The control 293FT/wild-type cell line demonstrated baseline activity of 7.037 ± 0.4 nmol hydrolyzed substrate/mL/h. Puromycin selection significantly enhanced enzymatic production in the engineered cells. Treatment with 1 µg/mL puromycin resulted in 329.7 ± 31.02 nmol/mL/h of GCase activity, a 47-fold increase compared to control (*p* = 0.0002). Increasing the puromycin concentration to 5 μg/mL resulted in 459.5 ± 24.82 nmol/mL/h (a 62-fold increase compared to the wild-type cell line, *p* < 0.0001). The higher puromycin concentration induced a 1.4-fold increase compared to the low-dose condition (*p* = 0.0007) (Figure 1A).

When normalized per million cells, the pattern of enzymatic secretion was maintained. The L17_293FT_GBA_OPT_HP cell line treated with 1 and 5 μg/mL puromycin exhibited enzymatic activities of 215.4 ± 39.46 U GCase/10^6^ cells and 346.1 ± 26.27 U GCase/10^6^ cells, respectively. These values represent a 48.5-fold increase with low stringency (*p* = 0.0017) and a 78-fold increase with high stringency (*p* = 0.0001) relative to control, corroborating the effectiveness of the puromycin selection on enzymatic productivity (Figure 1B).

**Figure 2:**
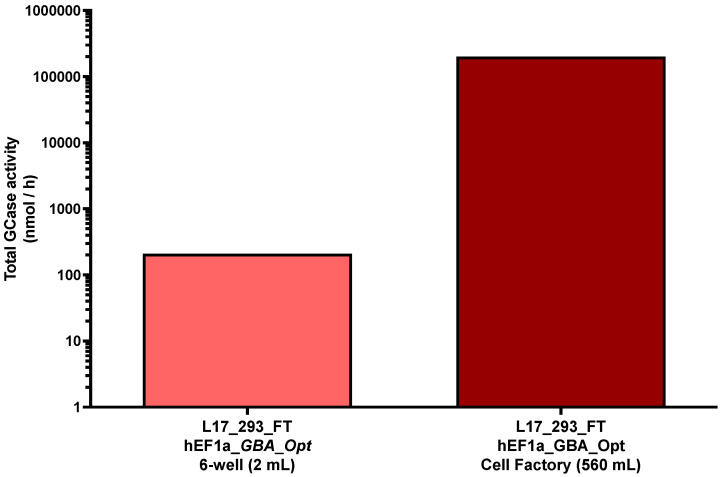
Total GCase production at different culture scales. Total β-glucocerebrosidase (GCase) activity (nmol/h) measured in the supernatant of 6-well plate (2 mL) and 10-layer cell factory system (560 mL) for the L17_293FT_GBA_OPT_HP cell line. “Total” refers to the absolute amount of enzyme produced per hour, calculated by multiplying the specific activity (nmol/mL/h) by the respective culture volume. A 963-fold increase in total enzyme production was observed upon scale-up, with preserved volumetric activity. Values are presented on a base-10 logarithmic scale. [293_FT = human embryonic kidney cells expressing SV40 large T antigen]; [hEF1α = human elongation factor 1-alpha promoter]; [GBA-Opt = codon-optimized human GBA (glucocerebrosidase) cDNA]; [GCase = β-glucocerebrosidase].

To evaluate scalability of the system, GCase production was assessed in a 10-layer cell factory and compared to that in small-scale cultures. The L17_293FT_GBA_OPT_HP cell line maintained consistent activity levels, producing 209.52 nmol/mL/h in a 6-well plate and 201,931 nmol/mL/h in the 10-layer system. This corresponded to a 280-fold increase in culture volume (from 2 mL to 560 mL), while preserving 96.4% of the volumetric enzymatic efficiency, resulting in a 963-fold increase in total GCase secretion (Figure 2).

**Figure 3:**
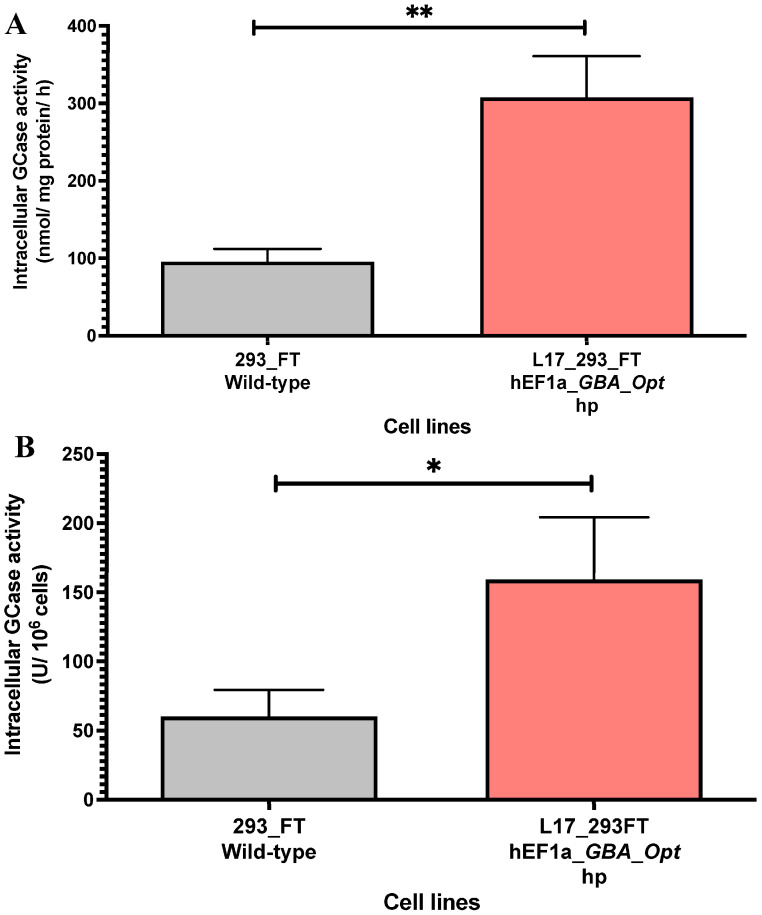
Intracellular GCase activity in L17_293FT_GBA_OPT_HP cells. (**A**) Intracellular GCase activity in cell lysates (nmol of hydrolyzed substrate per mg of total protein per hour) was significantly higher in the L17_293FT_GBA_OPT_HP population (307.5 ± 53.49 nmol/mg/h) compared to 293FT/wild-type controls (95.58 ± 16.5 nmol/mg/h; *p* = 0.0026; t = 7.572, df = 3.566; *n* = 4). (**B**) GCase activity normalized per million cells (U GCase/10^6^ cells) also showed enhanced production in the L17 population (159.3 ± 44.97 U/10^6^ cells) versus controls (60.2 ± 19.35 U/10^6^ cells; *p* = 0.0149; t = 4.048, df = 4.074; *n* = 4). Statistical analysis was performed using a two-tailed unpaired *t*-test with Welch’s correction. * indicates *p* ≤ 0.05; ** indicates *p* ≤ 0.01. [293_FT = human embryonic kidney cells expressing SV40 large T antigen]; [hEF1α = human elongation factor 1-alpha promoter]; [GBA-Opt = codon-optimized human GBA (glucocerebrosidase) cDNA]; [GCase = β-glucocerebrosidase]; [hp = puromycin-selected heterogeneous population].

The intracellular GCase activity was quantified in cell lysates from the L17_293FT_GBA_OPT_HP heterogeneous population. Compared to unmodified 293FT cells, the L17 population exhibited significantly higher enzymatic activity. The specific activity reached 307.5 ± 53.49 nmol/mg/h, which represents a 3.2-fold increase relative to control cells (95.58 ± 16.50 nmol/mg/h, *p* = 0.0026, Figure 3A). When normalized per million cells, the L17 population also demonstrated enhanced intracellular GCase production, reaching 159.3 ± 44.97 U GCase/10^6^ cells, compared to 60.2 ± 19.35 U GCase/10^6^ cells in control cells, a 2.64-fold increase relative to the control (*p* < 0.05; t = 4.048; df = 4.074, Figure 3B). These findings confirm that the selected population supports increased intracellular GCase secretion following lentiviral transduction and selection.

### 2.3. High-Producer Clone Selection from Puromycin-Selected Population

Eleven single-cell clones were isolated from the L17_293FT_GBA_OPT_HP heterogeneous population previously selected with 2 μg/mL puromycin. GCase secretion analysis revealed a broad range of enzymatic activities among clones, indicating variable transgene expression and productivity (Table 1). Secreted GCase levels ranged from 89.9 to 683.9 nmol/mL/h, reflecting the inherent heterogeneity of lentiviral transduced human cell populations.

Approximately 27% of the clones (3/11: CL13, CL15, CL16) exhibited high GCase secretion levels (>400 nmol/mL/h), 45% (5/11: CL5, CL8, CL9, CL11, CL17) showed moderate production (200–400 nmol/mL/h), and 28% (3/11: CL7, CL10, CL18) demonstrated low secretion (<200 nmol/mL/h).

Among them, clones 15 and 16 showed the highest enzymatic activities, with 585.4 and 683.9 nmol hydrolyzed substrate/mL/h, respectively, corresponding to 1.8-fold and 2.1-fold increases relative to the heterogeneous parental population. When normalized to cell number, these clones maintained their superior productivity, reaching 390.3 and 455.9 U/10^6^ cells, respectively (Table 1). These results confirm the successful enrichment of high GCase-producing cells through puromycin selection and single-cell cloning.

**Figure 4:**
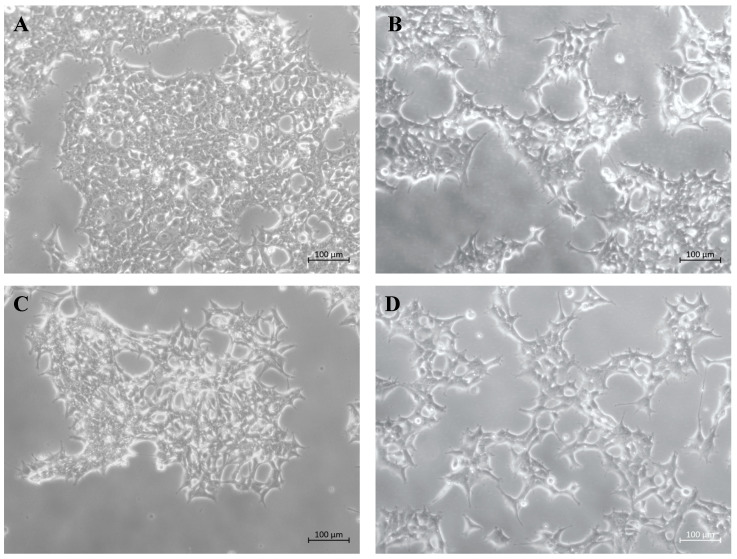
Morphological analysis of 293FT/wild-type and transgenic cell lines. (**A**) 293FT/wild-type cells showing characteristic fibroblast-like morphology; (**B**) Heterogeneous population L17_293FT_GBA_OPT_HP after 96 h in culture (3 × 10^5^ cells plated in a 25 cm^2^ flask); (**C**) Clone 15 (L17_293FT_GBA_OPT_CL15) after 120 h in cell culture (5 × 10^4^ cells plated in a 9.6 cm^2^ well); (**D**) Clone 16 (L17_293FT_GBA_OPT_CL16) after 24 h in culture (3 × 10^5^ cells plated in a 25 cm^2^ flask). Microscopy images were acquired using a ZEISS Axio inverted microscope with 10 × objective, and a 60N-C 1” 1.0 × Axiocam 503 color digital camera (ZEISS, Gottingen, Germany). Scale bar = 100 μm.

Morphological analysis by light microscopy revealed that a heterogeneous population (Figure 4B) and clones 15 and 16 (Figure 4C,D) retained a fibroblast-like morphology similar to wild-type cells (Figure 4A). Minor differences were observed and appeared to be related to cell density and culture confluence rather than to the transgene expression.

**Figure 5:**
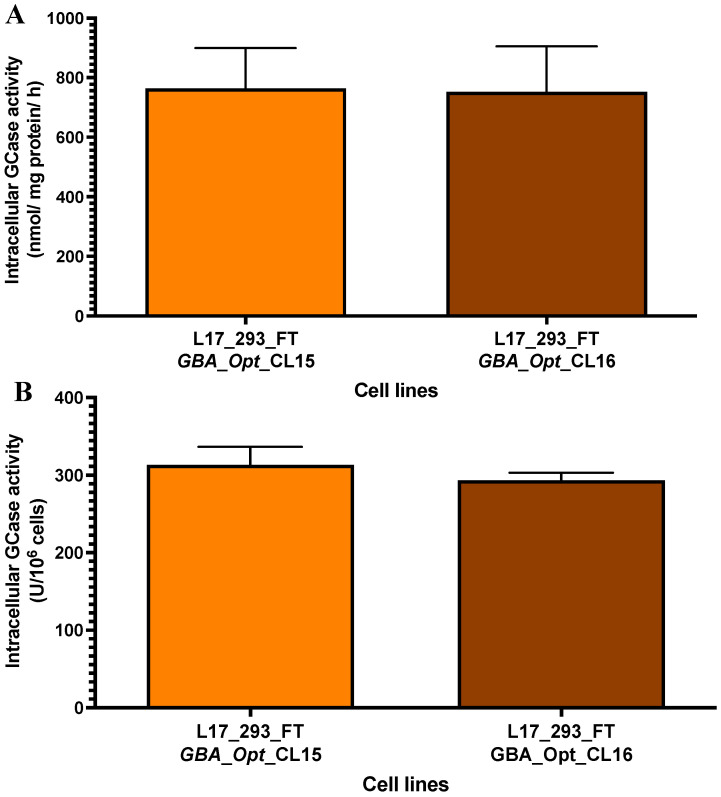
Intracellular GCase activity in L17-derived clones 15 and 16. (**A**) Intracellular GCase activity in cell lysates (nmol of hydrolyzed substrate per mg of total protein per hour) was comparable between clone 15 (763.8 ± 135.1, *n* = 3) and clone 16 (752.0 ± 152.1, n = 4), with no significant difference (*p* = 0.9292; t = 0.106, df = 4.749; *n* = 3 and n = 4). (**B**) GCase activity normalized per million cells (U GCase/10^6^ cells) was also similar between clone 15 (313.4 ± 13.4, *n* = 3) and clone 16 (293.3 ± 4.9, *n* = 4). Statistical analysis was performed using a two-tailed unpaired *t*-test with Welch’s correction. ns = not significant. [GCase = β-glucocerebrosidase]; [GBA_Opt_CL15 and CL16 = codon-optimized GBA-expressing single-cell clones derived from the L17_293FT_GBA_OPT line]. The number of replicates (n) for each clone is indicated in the figure legend.

To further characterize the two clones with the highest levels of secreted GCase (clones 15 and 16), we evaluated their intracellular GCase-specific activity. Clone 15 exhibited a specific activity of 763.8 ± 135.1 nmol of hydrolyzed substrate per mg of protein per hour, while clone 16 displayed a comparable activity of 752.0 ± 152.1 nmol/mg/h (Figure 5A). Compared to the L17_293FT_GBA_OPT_HP heterogeneous population, these values represent 2.5-fold (*p* = 0.01) and 2.4-fold (*p* = 0.006) increases, respectively. No statistically significant difference was observed between the two clones (*p* = 0.92). When compared with the 293FT/wild-type control cells, clone 15 exhibited a 7.9-fold increase (*p* = 0.01), and clone 16 a 7.8-fold increase (*p* = 0.003), corroborating with the high intracellular production of GCase in both clones. We also normalized GCase production per million cells. Clone 15 reached 313.4 ± 13.4 U GCase/10^6^ cells, and clone 16 reached 293.3 ± 4.9 U GCase/10^6^ cells (Figure 5B). These correspond to 1.96-fold and 1.84-fold increases compared to the L17 heterogeneous population (*p* = 0.0026 and *p* = 0.0078, respectively). Compared to the 293FT/wild-type control cells, clone 15 showed a 5.2-fold increase (*p* = 0.0001), and clone 16 showed a 4.8-fold increase (*p* < 0.0001).

## 3. Discussion

In this study, we established a stable human cell line capable of producing high levels of recombinant and synthetic human β-glucocerebrosidase (GCase). By integrating a codon-optimized *GBA1* gene (GBA-Opt) under the hEF1α promoter into 293FT cells via lentiviral vectors (LVs), we achieved sustained GCase specific activity up to 763.8 ± 135.1 nmol of hydrolyzed substrate per mg of protein per hour in a clonal population. This approach not only addresses the cost and scalability limitations of current ERT production systems but also demonstrates the potential applicability of synthetic biology to optimized therapeutic enzyme manufacture.

Lentiviral vectors (LVs) were selected for their ability to transduce both dividing and non-dividing cells and their preferential integration into transcriptionally active euchromatin regions, thereby enhancing transgene stability [67]. These key features were comprehensively described by Naldini in 2016 [67], and more recently reviewed in 2025, highlighting the continued evolution of LV platforms and their central role in advancing gene therapy for multiple genetic diseases [68]. The clinical relevance of LVs has been demonstrated by recent clinical trials and preclinical studies demonstrating efficacy in several monogenic and lysosomal storage disorders. Srivastava et al. [41] showed that CD34+ hematopoietic stem cells transduced with LVs carrying a codon-optimized FVIII gene yielded sustained expression in patients with hemophilia A. Similarly, Hu et al. [55] reported the successful preclinical application of SIV-LVs encoding IL2RG for X-linked severe combined immunodeficiency (X-SCID), confirming both efficacy and biosafety.

In the context of lysosomal diseases, Ellison et al. [58] developed and validated a GMP-grade LV manufacturing platform for hematopoietic stem cell gene therapy targeting MPS II, demonstrating process scalability, regulatory compliance, and preserved vector efficacy. Dogan et al. [59] used lentiviral delivery of engineered alpha-glucosidase (GAA) transgenes to improve secretion and tissue targeting in Pompe disease. These vectors conferred GAA enzymatic activity in both hematopoietic and target tissues, including muscle and CNS, highlighting the impact of vector engineering and transduction strategy. Mangiameli et al. [57] utilized LV-modified iPSC-derived neurons to model globoid cell leukodystrophy, illustrating the utility of LVs in disease modeling and potential autologous gene therapy. Finally, Saleh et al. [56] conducted a phase I trial in five patients with Fabry disease and demonstrated that lentiviral gene therapy resulted in persistent α-galactosidase secretion from transduced cells. Notably, three patients discontinued enzyme replacement therapy and maintained clinical stability, suggesting that LV-based delivery may enable long-term therapeutic enzyme production [56]. Collectively, these studies support broad therapeutic applicability of LV platforms and reinforce their suitability for GCase production.

It is worth noting that, our third-generation SIN LV efficiently delivered a large dual-cassette construct (13.5 kb) encoding GBA-Opt and puromycin resistance, achieving a high titer of 7.88 × 10^8^ VP/mL despite the near-maximal packaging capacity [69,70]. This performance likely reflects optimized 293FT packing cells, which express the SV40 large T antigen to enhance transfection efficiency [71,72,73,74]. Recent advances have further expanded the utility of this cell line enabling innovative delivery strategies such as dual-pseudotyped LVs (VSV-G/SeV-HN) for enhanced tropism and transduction efficiency in hematopoietic stem cells [75] and for non-integrating lentivirus-likely particles (Gag-only LVLPs) for safer delivery of base editors in cancer immunotherapy [76].

In our previous study [46], we demonstrated the feasibility of transient expression of codon-optimized *GBA1* (GBA-Opt) under the hEF1α promoter in 293FT cells. This construct led to a statistically significant 1.89-fold increase in relative mRNA expression compared to the CMV promoter (*p* = 0.001). Importantly, this transcriptional enhancement was accompanied by increased enzymatic activity: cells expressing GBA-Opt under hEF1α exhibited a mean intracellular GCase activity of 426.2 ± 25.1 nmol/mg/h, compared to 277.9 ± 17.5 nmol/mg/h with CMV, representing a ~1.5-fold increase [46]. Building on these observations and based on its consistent efficacy and stability across multiple human cell lines [77,78], we selected the hEF1α promoter. Kim et al. [32] demonstrated hEF1α-driven lentiviral vectors expressing GCase achieved higher expression levels than CMV-driven vector in HEK293, SH-SY5Y, and HeLa cells. In our system, lentiviral transduction with an hEF1α-driven GBA-Opt construct, followed by puromycin selection, led to stable integration into the host genome and resulted in high and sustained recombinant and synthetic GCase.

In parallel, codon optimization was applied to further enhance transgene expression. Our construct was designed with a codon-optimized *GBA1* sequence, aiming to improve mRNA stability and protein expression, as supported by prior studies showing that GC3 codons increase mRNA stability and protein levels in human cells [35]. This strategy has been widely used to optimize therapeutic gene expression in genetic diseases as demonstrated in hemophilia A trials [41] and SCID-X1 preclinical models [42]. The same strategy has also been successfully applied in several lysosomal storage diseases. Codon optimization of aspartylglucosaminidase (AGA) led to a 2.5 to 5-fold increase in expression in HEK293 and HeLa cells, supporting its utility for enzyme replacement therapy and gene therapy in aspartylglucosaminuria [43]. In globoid cell leukodystrophy, codon optimization of galactocerebrosidase (GALC) enabled safe and effective HSPC gene therapy in murine models [50]. The same group later reported long-term benefit in clinical trials using a similar lentiviral strategy for metachromatic leukodystrophy, reinforcing codon optimization as a key component in lysosomal gene therapy [79]. In mucopolysaccharidosis type II, a codon-optimized iduronate-2-sulfatase (IDS) construct driven by the MNDU3 promoter achieved supraphysiological enzyme levels across tissues and partial restoration in the brain, normalizing glycosaminoglycan (GAG) accumulation and preventing the emergence of cognitive deficits in vivo [48]. Expanding on this strategy, Liang et al. fused IGF2 to GAAco to enhance uptake via the CI-M6P/IGF2 receptor, enabling full correction of cardiac, skeletal, and CNS pathology at lower vector doses, thus identifying IGF2.GAA as a promising candidate for clinical translation [53]. A follow-up study by the same group confirmed that LV-IGF2.GAAco nearly normalized the skeletal muscle proteome in preclinical Pompe disease model without inducing off-target effects, reinforcing the therapeutic potential of codon-optimized LVs for lysosomal disorders [80].

While these studies underscore the translational relevance of codon-optimized constructs, we also evaluated the scalability potential of our production system. In cultures of 500 mL (6 × 10^8^ cells), the heterogeneous L17_293FT_GBA-Opt_HP population produced 201,931 nmol/mL/h of GCase, approximately a 963-fold increase over 2 mL cultures. The 10-layer cell factory run further confirmed this scalability, preserving over 96% of enzymatic efficiency observed in small-scale wells. The observed productivity likely reflects the combined effects of codon optimization, stable lentiviral, and puromycin selection of high-producing cells. In a previous study from our group, Rosa et al. [81] similarly reported enhanced recombinant factor VIII activity using SK-HEP cells cultured on microcarriers in spinner flasks, compared to static conditions. Although, codon optimization was not applied in that study, their findings highlight how optimized microcarrier-based systems can substantially enhance recombinant protein yields, serving as a complement to molecular engineering strategies such as those used in our work. While our current system utilized 10% FBS, the high levels of GCase production levels suggest promising potential for adaptation to serum-free conditions, an important consideration for future GMP-compliant production. Studies by Gomes-Alves et al. and Vicent et al. [82,83] have demonstrated the successful implementation of stirred microcarrier-based bioprocesses for large-scale, GMP-compatible production of human cells and recombinant proteins. These platforms offer valuable frameworks for scaling up production while ensuring quality attributes required for clinical translation. Importantly, the more recent work by Vicente et al. [83] further optimized oxygen control parameters to enhance both yield and functionality in stirred-tank bioreactors, reinforcing the feasibility of serum-free, high-density culture systems.

To enhance productivity, we tested increasing concentrations of puromycin under selective stringency. The L17_293FT_GBA_OPT_HP cell line, maintained in 10% FBS, exhibited GCase activity of 329.7 ± 5.51 nmol/mL/h and 439.5 ± 12.03 nmol/mL/h after treatment with 1 and 5 μg/mL puromycin, respectively. This dose-dependent increase in enzyme secretion was as much as 62-fold compared to the wild-type control and is consistent with previous findings that high-stringency drug selection enriches for integration events and transcriptional contexts permissive to strong transgene expression [60].

The L17 heterogeneous population, generated after six cycles of lentiviral transduction at MOIs of 30–50, showed a 3.2-fold increase in GCase activity compared to non-transduced cells. This enhancement aligns with Spencer et al. [84], who demonstrated that repeated transduction enhances recombinant enzyme output. Clonal selection further improved performance: among 11 clones analyzed, clones 15 and 16 reached 763.8 ± 135.1 nmol/mg/h and 752.0 ± 152.1 nmol/mg/h, representing up to an 8-fold increase over the parental cell line.

### Limitations and Future Directions

This study demonstrates the feasibility of using codon-optimized lentiviral system to generate high levels of recombinant GCase in human cells. Nonetheless, certain limitations must be acknowledged. A critical question that remains is whether the recombinant enzyme can be efficiently internalized by target cells via the mannose-6-phosphate receptor pathway, a prerequisite for appropriate lysosomal delivery and therapeutic action. While substantial in vitro activity was observed, the bioavailability and functional impact of the enzyme in disease-relevant settings remains to be established.

Moreover, the current system was specifically optimized for large-scale enzyme production in vitro. Its potential for gene therapy applications requires further investigation, particularly with respect to biosafety, durability of expression, and biodistribution in vivo.

To address these outstanding challenges, ongoing studies are evaluating cellular uptake, lysosomal targeting, and therapeutic efficacy of the recombinant and synthetic GCase in patient-derived fibroblasts and preclinical models of Gaucher disease. These efforts will help delineate the translational potential of this platform and its applicability to both enzyme replacement and gene-based therapies for lysosomal storage disorders.

## 4. Materials and Methods

### 4.1. Plasmid Constructs

The lentiviral expression vector used in this study was derived from pDEST_R4_R2 and contained a codon-optimized human *GBA1* cDNA, including the R534H missense mutation previously described [46]. The production of the lentiviral particles was performed using two accessory plasmids, pCMV-VSV-G (5824 bp) and pCMV△R8.91 (12,120 bp), kindly provided by Dr. Lucas Eduardo Botelho de Souza (Laboratory of Gene Transfer, Blood Center of Ribeirão Preto, SP, Brazil).

### 4.2. Cell Culture

The 293FT human cell line was purchased from Thermo, Waltham, MA, USA (R700-07). Cells were maintained in DMEM (Dulbecco modified Eagle’s medium) supplemented with 10% fetal bovine serum (FBS) (HyClone, South Logan, UT, USA), 1% penicillin/streptomycin/L-glutamine (Sigma-Aldrich, St. Louis, MO, USA), and 1% non-essential amino acids (HyClone). The serum was heat-inactivated at 56 °C for 30 min prior to use. Cell cultures were maintained at 37 °C in a humidified incubator with a 5% CO_2_ atmosphere. Cell growth was monitored every two days by phase-contrast microscopy, and the medium was refreshed as needed.

### 4.3. Production of Lentiviral Particles

To generate lentiviral particles, the construct DNA (pLV-hEF1a-GBA-Opt [46]) was transiently introduced into 293FT cells by triple co-transfection using lipofectamine (Life Technologies, Carlsbad, CA, USA) following the manufacturer’s instructions. The transfection mix included 6.5 μg of plasmid pCMV△R8.9, 3.2 μg of pCMV-VSV-G and 12 μg of the expression vector coding the GBA-Opt cDNA. Lentiviral supernatants were collected 48 h and 72 h post-transfection, filtered through a 0.22 μm Millex-GV filter (Millipore, Billerica, MA, USA), and concentrated by ultracentrifugation (1.40 h at 31,000× *g*) using an Optimat^TM^XL-100K ultracentrifuge (Beckman Coulter, Palo Alto, CA, USA) with a SW28 rotor, as previously described by [85,86]. The concentrated virus was stored at −80 °C. The viral titration was calculated by absolute quantification using real time PCR with the TaqMan system (Applied Biosystems, Foster City, MA, USA). The endogenous control was human β-actin gene (Hs03023880_g1), and lentiviral genome copies were quantified using primers and a probe targeting the LTR sequence: forward primer (5’-GCCCGAACAGGGACTTGA-3’), reverse primer (5’-CGAGTCCTGCGTCGAGAGA-3’) and the probe (5’-FAM-AGCGAAAGGGAAACC-MGB-3’). The viral titer (VP/mL) was calculated as described by [87], using the following formula: {[(Average LTR copy number × 2/Average β-actin copy number) × cell plated number] × dilution factor}. The lentiviral transductions were subsequently carried out at a multiplicity of infection (MOI) ranging from 30 to 50.

### 4.4. Lentiviral Transduction and Establishment of Stable Transduced Cell Lines

Stable cell populations expressing the codon-optimized *GBA1* (GBA-Opt) were generated by six consecutive rounds of lentiviral transduction in 293FT cells, using MOIs ranging from 30 to 50. For each round, 2 × 10^5^ cells were seeded in 6-well plates (2 mL DMEM per well). After 8 h, the medium was replaced with Opti-MEM (Gibco) and lentiviral particles were added in volume calculated based on viral tier (VP/mL) and desired MOI. Polybrene (6 μg/mL; Sigma-Aldrich) was added to enhance transduction efficiency. Cells were subjected to centrifugation (spinoculation) at 1200 rpm for 40 min at 22 °C. The following day, the medium was replaced with DMEM containing 10% FBS and 1% penicillin/streptomycin. Ater recovery and expansion, cells were trypsinized and replated for the next round of transduction. The interval between transduction cycles ranged from 2 to 7 days. Aliquots of transduced cells were cryopreserved between cycles to ensure experimental reproducibility.

### 4.5. Puromycin Treatment of L17_293FT_GBA_OPT_HP Heterogeneous Population

After the generation of the L17_293FT_GBA_OPT_HP heterogeneous population through 10 days of selection with 2 μg/mL of puromycin, cells were further exposed to 1 µg/mL or 5 μg/mL puromycin for 5 additional days to investigate the effect of different selection stringencies on GCase production. Following treatment, cells were expanded in T-75 flasks for enzymatic assays.

To ensure consistent selection pressure and maintain the expression of the integrated transgene, all experimental procedures involving this population, including scale-up studies, were preceded by a standardized 5-day puromycin treatment at 2 μg/mL. This approach was adopted across all assays involving L17_293FT_GBA_OPT_HP cells.

### 4.6. GCase Activity Analysis: Secreted and Intracellular (GCase-Specific Activity)

For enzymatic activity of secreted GCase activity, 3 × 10^5^ cells were seeded per well in 6-well plates and cultured for 48 h in DMEM supplemented with 10% FBS. The medium was then replaced for serum-free DMEM, and cells were incubated for an additional 48 h. At the end of this period, supernatants were collected, centrifuged at 1500 rpm for 5 min at 10 °C to remove cellular debris, and stored at −80 °C until enzymatic analysis.

For intracellular GCase activity, 2 × 10^5^ cells were seeded in 6-well plates and cultured in DMEM with 10% FBS and 1% antibiotics. After 48 h, the medium was replaced with fresh DMEM containing 10% FBS and 1% antibiotics. Following additional 48 h, cell pellets were collected in 245 μL of mammalian protein extraction buffer (GE Healthcare Life Sciences, Piscataway, NJ, USA), supplemented with 5 μL of protease inhibitor cocktail (Sigma-Aldrich). Lysates were homogenized and stored at −80 °C for subsequent analysis.

### 4.7. Scaling of GCase Production in L17_293FT_GBA_OPT_HP Cell Supernatants

For scale-up, 2 × 10^7^ cells from L17_293FT_GBA_OPT_HP population were seeded in 10-layer cell culture multi-flask (Corning, Kennebunk, ME, USA) and cultured for 72 h in 560 mL of DMEM containing 10% FBS and 1% antibiotic. After this period, the medium was replaced with serum-free DMEM, and cells were maintained for an additional 48 h. The supernatants were then harvested for enzymatic activity analysis. Cell pellets were collected by centrifugation at 2000 rpm, for 10 min at 10 °C and used for total cell count.

Total GCase activity (nmol/h) was calculated by multiplying the volumetric enzymatic activity (nmol/mL/h) by the total culture volume (2 mL for 6-well plates and 560 mL for the cell factory). This approach reflects the absolute amount of enzyme produced per hour, rather than its concentration, and was used to compare production yields at different culture scales.

### 4.8. Clone Cell Selection (Isolation)

Following the selection of the heterogeneous populations with 2 μg/mL puromycin, cells were expanded and 1 × 10^3^ transduced cells were seeded in 100 mm culture dishes to allow for single-cell clonal expansion. After approximately one week, discrete clonal colonies became visible and were individually harvested by trypsinization and transferred to 6-well plates for expansion. Each clonal population was then treated with puromycin (2 μg/mL) for five additional days to ensure transgene expression stability. Expanded clones were cryopreserved in aliquots of 3 × 10^6^ cells per cryotube and stored at −80 °C for further analyses.

### 4.9. Biological Activity by Fluorimetric Assay

Lysosomal GCase activities were measured using the synthetic fluorescent substrate 4-methylumbelliferyl-β-D-glucopyranoside (4-MUG) in the presence of sodium taurodeoxycholate according to the method described by [88], adapted from [89], and previously described by our group [46].

Before the enzymatic assay, total protein was quantified using the Lowry method [90] with the DC^TM^ Protein Assay Kit (Bio-Rad, Hercules, CA, USA), following the manufacturer’s instructions. All samples, including leukocytes lysates, wild-type cells, and transduced cell lines, were homogenized by sonication (3 × 10 s at 60 W, on ice) to guarantee complete cell lysis and protein extraction. The protein concentration was determined using a standard curve generated with serial dilutions of bovine serum albumin (BSA; 1.4 mg/mL to 0.175 mg/mL).

Each enzymatic reaction was prepared in amber tubes with 30 μL of the sample (containing 40–60 μg of total protein), 50 μL of 20 mM 4-MUG substrate, and 20 μL of 2% sodium taurodeoxycholate in 1M citrate-phosphate buffer (pH = 5.0). Reactions were incubated at 37 °C for 2 h, with gentle agitation, then cooled on ice for 5 min and stopped with the addition of 2 mL of glycine-KOH buffer (0.25 M, pH 10.3). All reactions were performed in triplicate.

A 200 μL aliquot of each reaction was transferred to black 96-well microplates, and relative fluorescence was measured using a Cary Eclipse fluorescence spectrophotometer (Agilent, Wilmington, DE, USA) with excitation at 360 nm and emission at 450 nm. Fluorescence readings were corrected using blanks and quantified by interpolation against a 4-methylumbelliferone (4-MU) standard curve. The enzymatic activity, as described in the literature [88], was expressed as nmol of hydrolyzed substrate per mg of protein per 1h. As reference, leukocyte lysates from healthy donors were included, with expected values between 8.68 and 11.57 nmol/mg/h as previously established [88].

The biological activity of secreted GCase present in the cell culture supernatant was also evaluated. The reaction conditions were identical to those described for intracellular lysates, except that 30 μL of supernatant was directly added to the reaction mix without adjustment for protein concentration. As with intracellular activity, all reactions were performed in triplicate, and fluorescence values were corrected using blanks and interpolated from the 4-MU standard curve.

### 4.10. Statistical Analysis

Statistical analyzes were performed using the non-parametric *t*-test with Welch correction, with *p*-value set at 0.05. Graphs were generated using GraphPad Prism software version 8.00 (GraphPad Software, San Diego, CA, USA).

## Figures and Tables

**Table 1:** GCase activity in the supernatant of single-cell clones derived from L17_293FT_GBA_OPT cell line.

Sample	GCase Activity (nmol/mL/h)	GCase Activity (U/10^6^ Cells)
L17_293FT_GBA_OPT_CL5	265.087	252.464
L17_293FT_GBA_OPT_CL7	89.911	128.444
L17_293FT_GBA_OPT_CL8	230.045	135.320
L17_293FT_GBA_OPT_CL9	301.160	200.773
L17_293FT_GBA_OPT_CL10	118.907	72.065
L17_293FT_GBA_OPT_CL11	285.561	219.662
L17_293FT_GBA_OPT_CL13	440.955	275.597
L17_293FT_GBA_OPT_CL15	585.464	390.310
L17_293FT_GBA_OPT_CL16	683.952	455.968
L17_293FT_GBA_OPT_CL17	207.610	143.180
L17_293FT_GBA_OPT_CL18	167.931	108.342

## Data Availability

Data is contained within the article.

## Abbreviations

The following abbreviations are used in this manuscript:

**Abbreviation**	**Meaning**
CHO	Chinese hamster ovary cell
DMEM	Dulbecco’s modified Eagle’s medium
ERT	Enzyme replacement therapy
ELISA	Enzyme-linked immunosorbent assay
FBS	Fetal bovine serum
FDA	Food and Drug Administration
*GBA1*	Glucocerebrosidase, Glucosylceramidase beta 1
GCase	Glucocerebrosidase, Glucosylceramidase beta 1
GD	Gaucher disease
GlcCer	Glucosylceramide
293_FT	Human embryonic kidney 293 cells
HP	Heterogeneous population
HS	High stringency
ICGG	International Collaborative Gaucher Group
L17	Lineage 17
LS	Low stringency
LV	Lentiviral vector
LTR	Long terminal repeat
MOI	Multiplicity of infection
MTX	Methotrexate
4MU	4-methylumbiliferone
4MUG	4-methylumbiliferon-β-D-glucopyranoside
qPCR	Real-time quantitative PCR
SUS	Unified Health System (Sistema Único de Saúde)
TDC	Sodium taurodeoxycholate hydrate
